# Phenazine–naphthalene-1,5-diamine–water (1/1/2)

**DOI:** 10.1107/S1600536809049009

**Published:** 2009-11-21

**Authors:** Agnieszka Czapik, Maria Gdaniec

**Affiliations:** a Faculty of Chemistry, Adam Mickiewicz University, 60-780 Poznań, Poland

## Abstract

The asymmetric unit of the title compound, C_12_H_8_N_2_·C_10_H_10_N_2_·2H_2_O, contains one half-mol­ecule of phenazine, one half-mol­ecule of naphthalene-1,5-diamine and one water mol­ecule. The phenazine and naphthalene-1,5-diamine mol­ecules are located on inversion centers. The water mol­ecules serve as bridges between the naphthalene-1,5-diamine mol­ecules and also between the naphthalene-1,5-diamine and phenazine mol­ecules. The naphthalene-1,5-diamine and water mol­ecules are connected *via* N—H⋯O and O—H⋯N hydrogen bonds, forming a *T*4(2) motif. They are arranged into a two-dimensional polymeric structure parallel to (10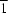) in which the water mol­ecule is a single donor and a double acceptor, whereas the amino group is a double donor and a single acceptor in the hydrogen bonding. These two-dimensional assemblies alternate with the layers of phenazine mol­ecules arranged into a herringbone motif. Each phenazine mol­ecule is hydrogen bonded to two water mol­ecules and thus a three-dimensional framework of hydrogen-bonded mol­ecules is generated.

## Related literature

For the structures of co-crystals of aromatic diaza­heterocycles with small aromatic mol­ecules, see: Thalladi *et al.* (2000 ▶); Kadzewski & Gdaniec (2006 ▶); Czapik & Gdaniec (2008 ▶). For structures with similar *T*4(2) hydrogen-bond motifs, see: Anthony *et al.* (2007 ▶); Neely *et al.* (2007 ▶). For symbols of hydrogen-bond motifs, see: Infantes *et al.* (2003 ▶). For a description of the Cambridge Structural Database, see: Allen (2002 ▶).
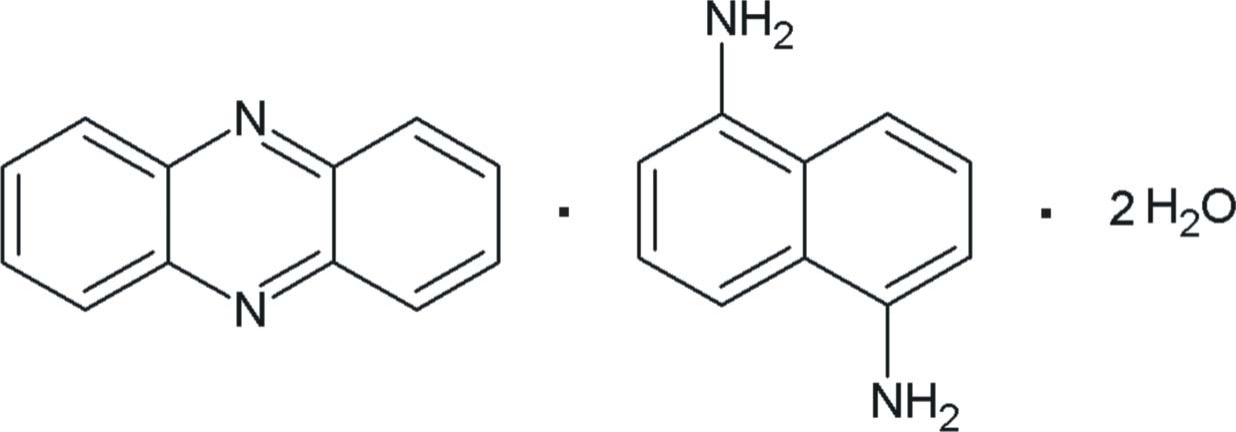


## Experimental

### 

#### Crystal data

C_12_H_8_N_2_·C_10_H_10_N_2_·2H_2_O
*M* *_r_* = 374.44
Monoclinic, 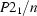

*a* = 13.0395 (10) Å
*b* = 4.9266 (2) Å
*c* = 15.7211 (12) Å
β = 112.508 (9)°
*V* = 933.00 (11) Å^3^
*Z* = 2
Mo *K*α radiation
μ = 0.09 mm^−1^
*T* = 130 K
0.25 × 0.25 × 0.25 mm

#### Data collection

Kuma KM-4-CCD κ-geometry diffractometer
Absorption correction: none
5251 measured reflections
1643 independent reflections
1357 reflections with *I* > 2σ(*I*)
*R*_int_ = 0.022

#### Refinement

*R*[*F* ^2^ > 2σ(*F* ^2^)] = 0.047
*wR*(*F* ^2^) = 0.140
*S* = 1.08
1643 reflections
143 parameters
H atoms treated by a mixture of independent and constrained refinement
Δρ_max_ = 0.22 e Å^−3^
Δρ_min_ = −0.23 e Å^−3^

### 

Data collection: *CrysAlis CCD* (Oxford Diffraction, 2007 ▶); cell refinement: *CrysAlis CCD*; data reduction: *CrysAlis RED* (Oxford Diffraction, 2007 ▶); program(s) used to solve structure: *SHELXS97* (Sheldrick, 2008 ▶); program(s) used to refine structure: *SHELXL97* (Sheldrick, 2008 ▶); molecular graphics: *ORTEP-3 for Windows* (Farrugia, 1997 ▶) and *Mercury* (Macrae *et al.*, 2006 ▶); software used to prepare material for publication: *SHELXL97*.

## Supplementary Material

Crystal structure: contains datablocks global, I. DOI: 10.1107/S1600536809049009/rz2392sup1.cif

Structure factors: contains datablocks I. DOI: 10.1107/S1600536809049009/rz2392Isup2.hkl

Additional supplementary materials:  crystallographic information; 3D view; checkCIF report

## Figures and Tables

**Table 1:** Hydrogen-bond geometry (Å, °)

*D*—H⋯*A*	*D*—H	H⋯*A*	*D*⋯*A*	*D*—H⋯*A*
N1*A*—H1*N*⋯O1*W*	0.91 (4)	2.10 (4)	2.999 (3)	169 (3)
N1*A*—H2*N*⋯O1*W* ^i^	0.97 (3)	2.15 (3)	3.102 (3)	166 (2)
O1*W*—H1*W*⋯N1*A* ^ii^	0.85 (5)	2.04 (5)	2.871 (3)	167 (4)
O1*W*—H2*W*⋯N1*B*	0.89 (3)	2.07 (3)	2.953 (3)	174 (3)

Symmetry codes: (i) 
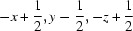
; (ii) 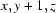.

**Fig. 1.**
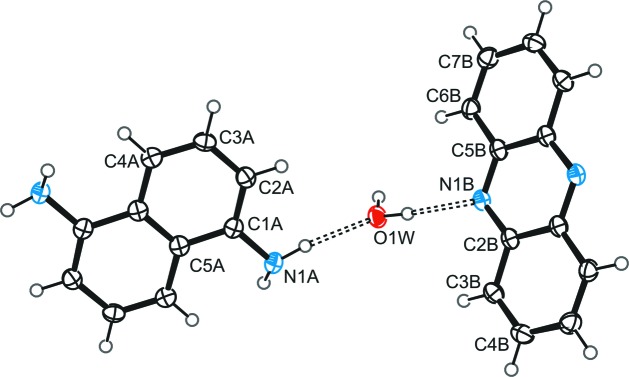
: The molecular structure of the title compound with displacement ellipsoids shown at the 50% probability level. Hydrogen bonds are shown as dashed lines and only atoms from the asymmetric unit are labelled.

**Fig. 2.**
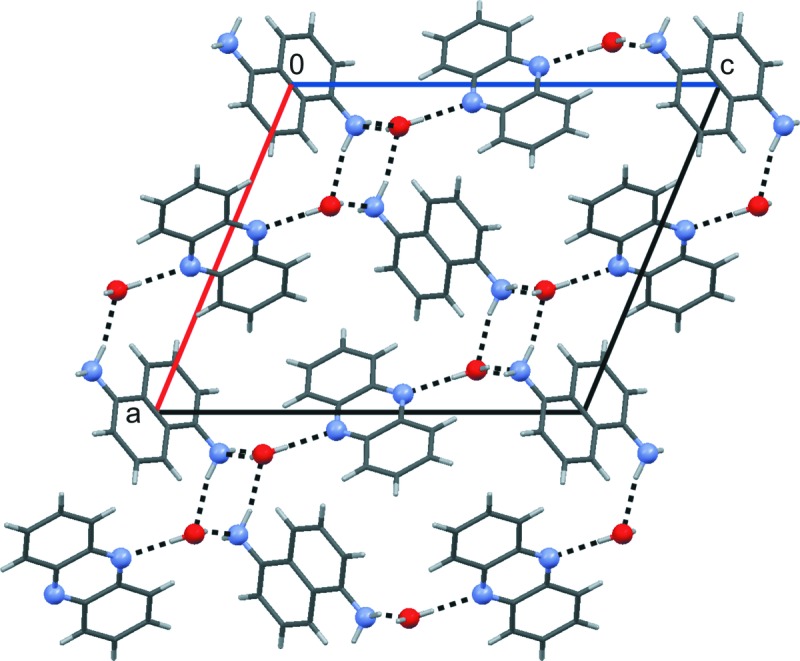
: Crystal packing viewed down the y axis. Hydrogen bonds are shown with dashed lines.

**Fig. 3.**
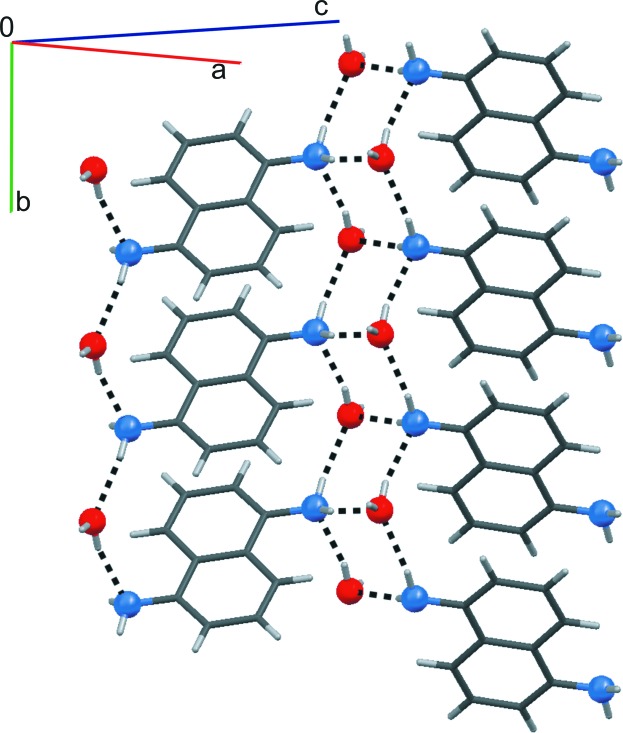
Hydrogen-bonded water molecule and aromatic amine generating the T4(2) motif.

**Fig. 4.**
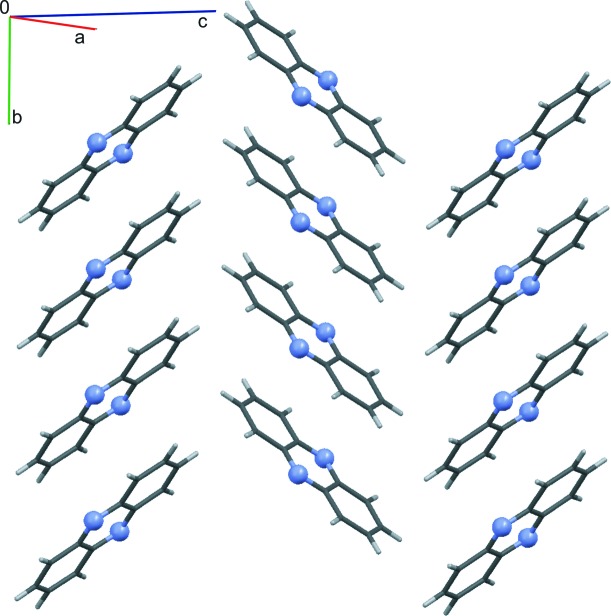
The herringbone arrangement of phenazine molecules parallel to (1 0 - 1)

## supplementary crystallographic information

### Comment

The title compound has been obtained unintentionally during our attempts to co-crystallize phenazine with naphthalene-1,5-diamine. Heterocycles like phenazine and quinoxaline are known to form a robust host framework with one-dimensional channels filled with small aromatic guest molecules (Thalladi *et al.*, 2000; Kadzewski & Gdaniec; 2006). Inclusion of water molecules have however a significant impact on arrangement of molecules in these co-crystals (Czapik & Gdaniec, 2008).

Crystal packing of the title compound is shown in Fig. 2. Phenazine and naphthalene-1,5-diamine molecules are situated around inversion centers and are arranged into stacks along [010] by π–π stacking interactions. The molecules of naphthalene-1,5-diamine and water are connected *via* N—H···O and O—H···N hydrogen bonds that form the T4(2) motif (Table 1, Fig. 3). These hydrogen bonds connect molecules into a two-dimensional polymeric structure parallel to (1 0 - 1) in which the water molecule is a single donor and a double acceptor whereas the amino group plays a role a double donor and a single acceptor (Fig. 3). The layers of naphthalene-1,5-diamine and water molecules alternate with the layers of phenazine in which these aromatic molecules show a herringbone arrangement (Fig. 4). The phenazine molecules are hydrogen bonded to two water molecules and thus a three-dimensional framework of hydrogen-bonded molecules is generated (Fig. 2).

The Cambridge Structural Database (Allen, 2002) was searched for the structures containing C—NH_2_ groups and water molecules to look for the frequency of the T4(2) motif (Infantes *et al.*, 2003) generated by primary amino groups and water molecules. The search was limited to organic compounds with polymeric and ionic structures excluded and gave only two structures with the CSD refcodes DISNEZ, (Anthony *et al.*, 2007) and MIMWAH01 (Neely *et al.*, 2007). In both cases the donor and acceptor functions of the amino group and water molecule were analogous to those in the title compound.

### Experimental

The title compound was obtained by dissolving phenazine (0.100 g, 0.55 mmol) and naphthalene-1,5-diamine (0.088 g, 0.55 mmol) in 5 ml of acetone. Slow evaporation of the solution yielded red cuboid crystals.

### Refinement

All H atoms were located in electron-density difference maps. C-bonded H atoms were placed at calculated positions, with C—H = 0.93 Å, and were refined as riding on their carrier C atoms, with *U*_ĩso_(H) = 1.2*U*_eq_(C). The H atoms of the OH and NH groups were freely refined (coordinates and isotropic displacement parameters).

### Crystal data

**Table:**

C_12_H_8_N_2_·C_10_H_10_N_2_·2H_2_O	*F*(000) = 396
*M**_r_* = 374.44	*D*_x_ = 1.333 Mg m^−^^3^
Monoclinic, *P*2_1_/*n*	Mo *K*α radiation, λ = 0.71073 Å
Hall symbol: -P 2yn	Cell parameters from 3369 reflections
*a* = 13.0395 (10) Å	θ = 2.6–27.9°
*b* = 4.9266 (2) Å	µ = 0.09 mm^−^^1^
*c* = 15.7211 (12) Å	*T* = 130 K
β = 112.508 (9)°	Cube, red
*V* = 933.00 (11) Å^3^	0.25 × 0.25 × 0.25 mm
*Z* = 2	

### Data collection

**Table:**

Kuma KM-4-CCD κ-geometry diffractometer	1357 reflections with *I* > 2σ(*I*)
Radiation source: fine-focus sealed tube	*R*_int_ = 0.022
graphite	θ_max_ = 25.0°, θ_min_ = 4.4°
ω scans	*h* = −15→15
5251 measured reflections	*k* = −5→5
1643 independent reflections	*l* = −18→18

### Refinement

**Table:**

Refinement on *F*^2^	Primary atom site location: structure-invariant direct methods
Least-squares matrix: full	Secondary atom site location: difference Fourier map
*R*[*F*^2^ > 2σ(*F*^2^)] = 0.047	Hydrogen site location: inferred from neighbouring sites
*wR*(*F*^2^) = 0.140	H atoms treated by a mixture of independent and constrained refinement
*S* = 1.08	*w* = 1/[σ^2^(*F*_o_^2^) + (0.0606*P*)^2^ + 1.1003*P*] where *P* = (*F*_o_^2^ + 2*F*_c_^2^)/3
1643 reflections	(Δ/σ)_max_ < 0.001
143 parameters	Δρ_max_ = 0.22 e Å^−^^3^
0 restraints	Δρ_min_ = −0.23 e Å^−^^3^

### Special details

**Table:**

Geometry. All e.s.d.'s (except the e.s.d. in the dihedral angle between two l.s. planes) are estimated using the full covariance matrix. The cell e.s.d.'s are taken into account individually in the estimation of e.s.d.'s in distances, angles and torsion angles; correlations between e.s.d.'s in cell parameters are only used when they are defined by crystal symmetry. An approximate (isotropic) treatment of cell e.s.d.'s is used for estimating e.s.d.'s involving l.s. planes.
Refinement. Refinement of *F*^2^ against ALL reflections. The weighted *R*-factor *wR* and goodness of fit *S* are based on *F*^2^, conventional *R*-factors *R* are based on *F*, with *F* set to zero for negative *F*^2^. The threshold expression of *F*^2^ > σ(*F*^2^) is used only for calculating *R*-factors(gt) *etc*. and is not relevant to the choice of reflections for refinement. *R*-factors based on *F*^2^ are statistically about twice as large as those based on *F*, and *R*- factors based on ALL data will be even larger.

### Fractional atomic coordinates and isotropic or equivalent isotropic displacement parameters (Å^2^)

**Table:**

	*x*	*y*	*z*	*U*_iso_*/*U*_eq_	
N1A	0.12558 (17)	0.2658 (5)	0.18906 (13)	0.0249 (5)	
H1N	0.116 (3)	0.419 (8)	0.218 (2)	0.054 (10)*	
H2N	0.198 (3)	0.258 (6)	0.1847 (19)	0.038 (8)*	
C1A	0.03659 (18)	0.2272 (5)	0.10357 (15)	0.0220 (5)	
C2A	−0.05954 (19)	0.3753 (5)	0.08078 (15)	0.0245 (5)	
H2A	−0.0655	0.5056	0.1214	0.029*	
C3A	−0.14918 (19)	0.3314 (5)	−0.00367 (16)	0.0248 (5)	
H3A	−0.2135	0.4343	−0.0185	0.030*	
C4A	0.14256 (19)	−0.1391 (5)	0.06406 (16)	0.0243 (5)	
H4A	0.2021	−0.1134	0.1197	0.029*	
C5A	0.04549 (18)	0.0218 (5)	0.04242 (15)	0.0223 (5)	
N1B	0.05503 (15)	0.9466 (4)	0.43964 (12)	0.0220 (5)	
C2B	0.08133 (18)	0.8115 (5)	0.51930 (15)	0.0211 (5)	
C3B	0.16597 (18)	0.6111 (5)	0.54398 (16)	0.0252 (5)	
H3B	0.2031	0.5753	0.5051	0.030*	
C4B	0.19301 (19)	0.4712 (5)	0.62413 (17)	0.0279 (6)	
H4B	0.2489	0.3411	0.6399	0.033*	
C5B	−0.02539 (18)	1.1344 (5)	0.41932 (15)	0.0217 (5)	
C6B	−0.0560 (2)	1.2869 (5)	0.33626 (15)	0.0258 (6)	
H6B	−0.0206	1.2553	0.2959	0.031*	
C7B	−0.1367 (2)	1.4781 (5)	0.31585 (16)	0.0290 (6)	
H7B	−0.1555	1.5778	0.2617	0.035*	
O1W	0.12857 (15)	0.7813 (4)	0.29145 (12)	0.0297 (5)	
H1W	0.137 (3)	0.932 (10)	0.269 (3)	0.074 (13)*	
H2W	0.107 (2)	0.819 (6)	0.337 (2)	0.035 (8)*	

### Atomic displacement parameters (Å^2^)

**Table:**

	*U*^11^	*U*^22^	*U*^33^	*U*^12^	*U*^13^	*U*^23^
N1A	0.0267 (11)	0.0260 (12)	0.0211 (10)	−0.0033 (9)	0.0081 (8)	−0.0025 (9)
C1A	0.0246 (12)	0.0221 (12)	0.0212 (11)	−0.0043 (10)	0.0108 (9)	0.0018 (9)
C2A	0.0294 (12)	0.0227 (12)	0.0247 (12)	−0.0004 (10)	0.0138 (10)	0.0008 (10)
C3A	0.0222 (11)	0.0244 (13)	0.0292 (12)	0.0022 (10)	0.0114 (10)	0.0043 (10)
C4A	0.0223 (11)	0.0243 (13)	0.0261 (12)	−0.0029 (10)	0.0089 (9)	0.0011 (10)
C5A	0.0261 (11)	0.0201 (12)	0.0240 (11)	−0.0036 (9)	0.0132 (10)	0.0020 (9)
N1B	0.0252 (10)	0.0201 (10)	0.0230 (10)	−0.0026 (8)	0.0119 (8)	−0.0032 (8)
C2B	0.0226 (11)	0.0181 (12)	0.0247 (11)	−0.0044 (9)	0.0114 (9)	−0.0038 (9)
C3B	0.0245 (12)	0.0240 (12)	0.0302 (12)	−0.0001 (10)	0.0139 (10)	−0.0017 (10)
C4B	0.0250 (12)	0.0216 (13)	0.0346 (13)	0.0027 (10)	0.0085 (10)	−0.0008 (10)
C5B	0.0228 (11)	0.0196 (12)	0.0246 (12)	−0.0039 (9)	0.0111 (9)	−0.0040 (9)
C6B	0.0312 (12)	0.0262 (13)	0.0220 (12)	−0.0006 (11)	0.0124 (10)	−0.0005 (10)
C7B	0.0355 (13)	0.0237 (13)	0.0257 (12)	−0.0014 (11)	0.0094 (10)	0.0021 (10)
O1W	0.0405 (10)	0.0279 (11)	0.0257 (9)	0.0042 (8)	0.0182 (8)	−0.0001 (8)

### Geometric parameters (Å, °)

**Table:**

N1A—C1A	1.412 (3)	N1B—C5B	1.342 (3)
N1A—H1N	0.91 (4)	C2B—C3B	1.420 (3)
N1A—H2N	0.97 (3)	C2B—C5B^ii^	1.440 (3)
C1A—C2A	1.374 (3)	C3B—C4B	1.359 (3)
C1A—C5A	1.431 (3)	C3B—H3B	0.9300
C2A—C3A	1.410 (3)	C4B—C7B^ii^	1.422 (4)
C2A—H2A	0.9300	C4B—H4B	0.9300
C3A—C4A^i^	1.367 (3)	C5B—C6B	1.425 (3)
C3A—H3A	0.9300	C6B—C7B	1.356 (3)
C4A—C3A^i^	1.367 (3)	C6B—H6B	0.9300
C4A—C5A	1.420 (3)	C7B—H7B	0.9300
C4A—H4A	0.9300	O1W—H1W	0.85 (5)
C5A—C5A^i^	1.422 (4)	O1W—H2W	0.89 (3)
N1B—C2B	1.341 (3)		
			
C1A—N1A—H1N	111 (2)	N1B—C2B—C3B	119.61 (19)
C1A—N1A—H2N	113.2 (16)	N1B—C2B—C5B^ii^	121.3 (2)
H1N—N1A—H2N	113 (3)	C3B—C2B—C5B^ii^	119.1 (2)
C2A—C1A—N1A	120.8 (2)	C4B—C3B—C2B	120.3 (2)
C2A—C1A—C5A	120.1 (2)	C4B—C3B—H3B	119.8
N1A—C1A—C5A	119.1 (2)	C2B—C3B—H3B	119.8
C1A—C2A—C3A	120.6 (2)	C3B—C4B—C7B^ii^	120.7 (2)
C1A—C2A—H2A	119.7	C3B—C4B—H4B	119.7
C3A—C2A—H2A	119.7	C7B^ii^—C4B—H4B	119.7
C4A^i^—C3A—C2A	120.7 (2)	N1B—C5B—C6B	120.1 (2)
C4A^i^—C3A—H3A	119.7	N1B—C5B—C2B^ii^	121.2 (2)
C2A—C3A—H3A	119.7	C6B—C5B—C2B^ii^	118.7 (2)
C3A^i^—C4A—C5A	120.5 (2)	C7B—C6B—C5B	120.2 (2)
C3A^i^—C4A—H4A	119.7	C7B—C6B—H6B	119.9
C5A—C4A—H4A	119.7	C5B—C6B—H6B	119.9
C4A—C5A—C5A^i^	119.2 (3)	C6B—C7B—C4B^ii^	121.0 (2)
C4A—C5A—C1A	121.9 (2)	C6B—C7B—H7B	119.5
C5A^i^—C5A—C1A	118.9 (3)	C4B^ii^—C7B—H7B	119.5
C2B—N1B—C5B	117.47 (18)	H1W—O1W—H2W	107 (3)

Symmetry codes: (i) −*x*, −*y*, −*z*; (ii) −*x*, −*y*+2, −*z*+1.

### Hydrogen-bond geometry (Å, °)

**Table:**

*D*—H···*A*	*D*—H	H···*A*	*D*···*A*	*D*—H···*A*
N1A—H1N···O1W	0.91 (4)	2.10 (4)	2.999 (3)	169 (3)
N1A—H2N···O1W^iii^	0.97 (3)	2.15 (3)	3.102 (3)	166 (2)
O1W—H1W···N1A^iv^	0.85 (5)	2.04 (5)	2.871 (3)	167 (4)
O1W—H2W···N1B	0.89 (3)	2.07 (3)	2.953 (3)	174 (3)

Symmetry codes: (iii) −*x*+1/2, *y*−1/2, −*z*+1/2; (iv) *x*, *y*+1, *z*.
